# alleHap: an efficient algorithm to reconstruct zero-recombinant haplotypes from parent-offspring pedigrees

**DOI:** 10.1186/1471-2105-15-S3-A6

**Published:** 2014-02-11

**Authors:** Nathan Medina-Rodríguez, Angelo Santana, Ana M Wägner, José M Quinteiro

**Affiliations:** 1 Department of Mathematics, Universidad de Las Palmas de Gran Canaria, Campus de Tafira, 35017 Las Palmas, Spain; 2 IUMA - Information and Communication Systems, Universidad de Las Palmas de Gran Canaria, Campus de Tafira, 35017 Las Palmas, Spain; 3 Department of Medical and Surgical Sciences, Universidad de Las Palmas de Gran Canaria, Campus de Tafira, 35017 Las Palmas, Spain

## Background

Haplotype inference is an essential stage in genetic linkage analysis and estimation methods are also very frequently used to reconstruct haplotypes in current genetic association studies. Most of the latter are focused on haplotype phasing from recombinant DNA areas of unrelated individuals and use likelihood-based methods to infer the presence of alleles in several loci with very time-consuming probabilistic algorithms.

So far, literature does not analyze haplotypes using deterministic techniques, and there are hardly any alternative methods for constructing haplotypes from non-recombinant DNA areas, despite the fact that computational inference by probabilistic models may cause a large number of incorrect inferences.

## Description and results

We have developed an algorithm called alleHap, which is able to impute alleles from parent-offspring pedigree databases with missing family members, to later construct their corresponding, unambiguous haplotypes.

The alleHap algorithm is based on a preliminary analysis of all possible combinations that may exist in the genotyping of a family, considering that each member, due to meiosis, should unequivocally have two alleles, one from each parent. The analysis was founded on the differentiation of seven cases, as described in [1], but some of them divided into a maximum of three variants, representing a different combination of alleles of the family members (Table 1).

**Table 1:** Possible allelic combinations in a parent-offspring pedigree

Parents	1		2		Offspring^*^	1		2		3		4		Case
*Alleles*	*1*	*2*	*1*	*2*	*Alleles*	*1*	*2*	*1*	*2*	*1*	*2*	*1*	*2*	*(Variant)*
			
	a	a	a	a		a	a							1
	a	a	b	b		a	b							2
	a	a	a	b		a	a	a	b					3 *(I)*
	a	b	b	b		a	b	b	b					3 *(II)*
	a	a	b	c		a	b	a	c					4 *(I)*
	a	c	b	b		a	b	b	c					4 *(II)*
	a	b	c	c		a	c	b	c					4 *(III)*
	a	b	a	b		a	a	a	b	b	b			5
	a	b	a	c		a	a	a	b	a	c	b	c	6 *(I)*
	a	b	b	c		a	b	a	c	b	b	b	c	6 *(II)*
	a	c	b	c		a	b	a	c	b	c	c	c	6 *(III)*
	a	b	c	d		a	c	a	d	b	c	b	d	7 *(I)*
	a	c	b	d		a	b	a	d	b	c	c	d	7 *(II)*
	a	d	b	c		a	b	a	c	b	d	c	d	7 *(III)*

^*^Considering all allele combinations, the maximum number of “unique” children and alleles is four.

The classification by cases and variants allows the algorithm to impute missing values efficiently in the loaded database to proceed afterwards to the conformation of corresponding unambiguous haplotypes. Furthermore, the algorithm allows the construction of haplotypes, without any limitation in terms of the number of SNPs, i.e. enables the construction of haplotypes of more than two SNPs.

By analyzing all possible combinations of a parent-offspring pedigree in which parents may be missing, as long as one child has been genotyped, theoretically an unequivocal imputation of three possible parent haplotypes is possible in 92.3% of cases even when one parent is missing. When neither parent has been genotyped, in 36.4% of cases at least two haplotypes can be constructed. Regarding offspring allele imputation with both parents fully genotyped, a minimum of one haplotype for each child may be successfully reconstructed in 6.1% of possible cases.

**Figure 1:**
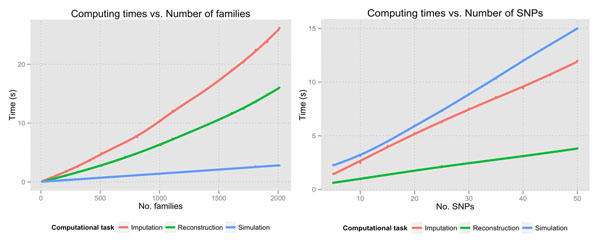
Representation of computing times according to the number of families (left) and the number of SNPs (right).

Evaluation of the results (Figure 1) reveals an optimum performance of alleHap computational tasks, namely Simulation, Imputation and Reconstruction. Their corresponding execution times are quite low even when considering a large number of families (≤ 2000) and SNPs (≤ 50).

**Figure 2:**
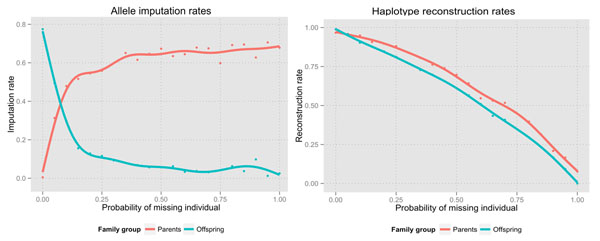
Representation of allele imputation rates (left) and haplotype reconstruction rates (right).

Figure 2 shows how our algorithm has high allele imputation rates (about 65%) even when the probability of missing parents in each family is high (>50%). Regarding haplotype reconstruction rates, there is an almost linear relationship between reconstruction rates and the number of missing individuals per family. This is because alleHap is mainly based on the information included in the offspring, so the more children that are missing the more difficult it is to reconstruct the family haplotypes.

## Conclusions

alleHap has been tested by simulations and also with the Type 1 Diabetes Genetics Consortium [2] database. Our algorithm is very robust against inconsistencies within the genotypic data and consumes very little time, even when handling large amounts of data. The missing data imputation may improve results in numerous epidemiological and/or genetic linkage studies.

Our algorithm could be a useful instrument for information retrieval and knowledge discovery in genetics, since it would allow epidemiological specialists to discover new intergenic patterns by studying zero-recombinant haplotypes with a larger number of SNPs from family-based databases.
